# Chloroplast Genome Characterization and Phylogenetic Implications of *Reyesia* and *Salpiglossis* (Salpiglossideae, Solanaceae)

**DOI:** 10.1002/ece3.74599

**Published:** 2026-10-27

**Authors:** Jie Huang, Suyun Nong, Yancheng Zhang, Weibin Xu

**Affiliations:** ^1^ Guangxi Key Laboratory of Plant Conservation and Restoration Ecology in Karst Terrain Guangxi Institute of Botany, Guangxi Zhuangzu Autonomous Region and Chinese Academy of Sciences Guilin China

**Keywords:** chloroplast genome, phylogenetic relationship, *Reyesia*, Salpiglossideae, *Salpiglossis*

## Abstract

Solanaceae represents one of the most economically significant families among flowering plants. Genomic research within the family has primarily concentrated on some crop genera, such as *Solanum* L., *Nicotiana* L., and *Capsicum* L., whereas early‐diverging clades remain comparatively understudied. This study presents the complete chloroplast (cp) genomes of two species belonging to the tribe Salpiglossideae (subfamily Cestroideae), *Reyesia chilensis* Clos and *Salpiglossis sinuata* Ruiz & Pav, and performs comprehensive comparative analyses across representative Solanaceae plastomes, focusing on genome structure, gene content, repeats, codon usage, nucleotide polymorphism (Pi), and phylogenetic relationships. The cp genomes of *R. chilensis* and *S. sinuata* have lengths of 156,969 and 156,262 bp, respectively, and both exhibit the characteristic quadripartite structure. Their genome organization and gene composition were highly conserved. The plastome of *R. chilensis* encoded 112 unique genes (111 in *S. sinuata* ), including 79 protein‐coding genes, 29 transfer RNA‐coding genes (28 in *S. sinuata* ), and four ribosomal RNA‐coding genes. Comparative analyses revealed only minor sequence divergence between the two plastomes, with highly similar codon usage bias and repeat sequence composition. Phylogenetic analyses supported *R. chilensis* and *S. sinuata* as a clade representing Salpiglossideae, sister to *Benthamiella patagonica* Speg. (Benthamielleae), together forming an early‐diverging branch within the Solanaceae. Nucleotide polymorphism analyses revealed a mean nucleotide diversity (Pi) of 0.013 within the shared genes, whereas intergenic spacers exhibited a higher sequence polymorphism (average Pi = 0.028). Several hypervariable segments were identified as promising markers for Solanaceae species delimitation and phylogenetic inference. This work contributes critical genomic datasets and further clarifies the phylogenetic comprehension across the family.

## Graphical abstract

We generated and compared the complete chloroplast genomes of *Reyesia chilensis* and *Salpiglossis sinuata* , two representative species of the tribe Salpiglossideae (subfamily Cestroideae) within Solanaceae. Comparative plastome analyses revealed highly conserved genome organization, codon usage, and repeat composition, while phylogenetic analyses supported Salpiglossideae as sister to *Benthamiella patagonica*, together forming the early‐diverging clade. Several highly variable coding and intergenic regions were identified as promising molecular markers for phylogenetic reconstruction and species identification in Solanaceae.

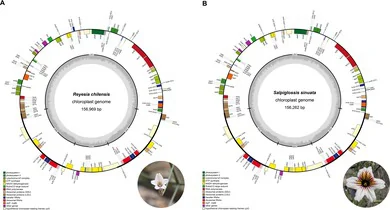

## 1 Introduction

Solanaceae represents a family of flowering plants with considerable economic value, containing ~3000 species distributed worldwide (Barboza et al. 2016). Phylogenetic studies of Solanaceae have progressed from analyses based on limited taxon sampling and only one or several chloroplast (cp) DNA regions to studies incorporating broader sampling across the family or a specific clade (subfamily/tribe/genus), and ultimately to contain representatives of nearly complete genus‐level sampling or phylogenomic datasets with massive molecular markers (Hawkes 1990; Bohs and Olmstead 1997; Olmstead et al. 1999, 2008; Knapp 2002a; Bohs 2005; Olmstead and Bohs 2006; Särkinen et al. 2013; Ng and Smith 2016; Gagnon et al. 2022; Huang, Xu, et al. 2023; Deanna et al. 2026). Through these successive advances, the phylogenetic framework of Solanaceae has gradually become increasingly well‐resolved.

Based on analyses of nearly 1700 nuclear loci, Huang, Xu, et al. (2023) resolved seven major subfamilies within the family, that is, Schizanthoideae, Goetzeioideae, Cestroideae, Schwenckioideae, Petunioideae, Nicotianoideae, and Solanoideae. Recently, Deanna et al. (2026) generated the most taxonomically comprehensive phylogeny of Solanaceae to date, incorporating 1474 species representing 100 of the 102 recognized genera and using molecular data from the Angiosperm353 target‐enrichment dataset together with plastid and nuclear markers. Their phylogenetic classification retained only those traditional taxonomic names that were strongly supported by phylogenetic evidence and introduced 38 rank‐free clade names to provide a more stable and informative nomenclatural framework (Deanna et al. 2026).

Despite these advances, genomic resources remain unevenly distributed across the family. Most comparative genomic and evolutionary studies have focused on economically important lineages, particularly members of *Solanum*, *Nicotiana*, and *Capsicum* (Wang et al. 2022, 2023; Yan et al. 2023; He et al. 2024). In contrast, several early‐diverging groups, including representatives of Cestroideae, Schizanthoideae, and other phylogenetically pivotal lineages, remain poorly sampled at the genomic level. The circumscription and systematic placement of Salpiglossideae in Solanaceae have long been subjects of taxonomic debate (D'Arcy 1978; Olmstead and Palmer 1992; Olmstead et al. 1999; Knapp 2002a, 2002b; Dillon 2005). Early classifications by Benth. (1835) defined the Salpiglossideae primarily on floral characteristics, particularly bilateral corolla symmetry and a reduction in stamen number, resulting in the inclusion of several genera, such as *Salpiglossis* Ruiz & Pav., *Reyesia* Clos, *Browallia* L., *Streptosolen* Miers, *Schizanthus* Ruiz & Pav., and *Brunfelsia* Plum. ex L. (D'Arcy 1978). However, subsequent molecular phylogenetic investigations demonstrated that this circumscription was non‐monophyletic (Olmstead and Palmer 1992; Fay et al. 1998; Olmstead et al. 1999). Based on a revised interpretation of morphological evidence, Hunziker (2001) restricted Salpiglossideae to two closely related genera, *Reyesia* and *Salpiglossis*, and placed them within subfamily Cestroideae. This circumscription was subsequently adopted in the Solanaceae monograph by Barboza et al. (2016), in which *Reyesia* was recognized as containing four species distributed primarily in Chile, whereas *Salpiglossis* included two species native to the southern Andes of Chile and Argentina.

*Reyesia* and *Salpiglossis* are morphologically similar but have varied phylogenetic positions across previous studies (Dillon 2005; Stafford and Knapp 2006; Olmstead et al. 2008; Särkinen et al. 2013; Ng and Smith 2016). Within the nuclear phylogenomic phylogeny, Salpiglossideae (comprising these two genera) was recovered, together with the other two tribes, Browallieae and Cestreae, forming the subfamily Cestroideae, which represents one of the early‐diverging major lineages within Solanaceae (Huang, Xu, et al. 2023). However, within the PhyloCode framework, Cestreae was the only rank‐free clade recognized in the subfamily Cestroideae, likely because the phylogenetic relationships among the remaining members of Cestroideae remain controversial (Deanna et al. 2026).

Chloroplast genomes are among the most widely used genomic resources in plant evolutionary research because of their generally conserved gene structure organization and uniparental inheritance. In most angiosperms, the plastome possesses a conserved quadripartite architecture consisting of a large single‐copy (LSC) region, a small single‐copy (SSC) region, and a pair of inverted repeat (IR) regions. Owing to these features, chloroplast genomes have become indispensable for studies of species classification, phylogeography, plant systematics, and conservation (Liu et al. 2018; Song et al. 2023). Furthermore, several highly variable plastid loci have been extensively employed as DNA barcodes and phylogenetic markers, providing important genetic information and resources for the reconstruction of phylogenetic frameworks in angiosperms (Chase et al. 2007; Hollingsworth et al. 2009; Li, Liu, et al. 2011).

In this study, we assembled and characterized the complete chloroplast genomes of *Reyesia chilensis* and *Salpiglossis sinuata* . These chloroplast genomes represent the first genomic resources available for *Reyesia* and *Salpiglossis*, the tribe Salpiglossideae, and even for the subfamily Cestroideae. The newly generated data fill an important gap in plastome sampling within Solanaceae and provide a valuable foundation for comparative plastome analyses, taxonomy, evolutionary, and conservation research in this diverse plant family.

## 2 Materials and Methods

### 2.1 Plant Materials and Sequencing

Leaf materials of *R. chilensis* and *S. sinuata* were collected from living plants cultivated at Guangxi Institute of Botany, Chinese Academy of Sciences (Guilin, China). Total genomic DNA from each sample was extracted following a modified CTAB method (Doyle and Doyle 1987). Purified DNA was randomly fragmented and used for library preparation with an insert size of approximately 300–400 bp. Paired‐end reads (150 bp) were generated on the DNBSEQ‐T7 platform at BGI (Wuhan, China). A total of around 10 GB of raw sequencing data was obtained. Raw reads were quality‐filtered using SOAPnuke (Chen et al. 2018), and only high‐quality reads were retained for downstream analyses.

### 2.2 Plastome Assembly and Genome Annotation

High‐quality reads of both species were *de novo* assembled into complete plastomes using GetOrganelle v1.7.6.1 (Jin et al. 2020). Assembly graphs were visualized in Bandage v0.8.1 (Wick et al. 2015) to confirm genome circularity. In addition, the original reads were remapped to the assembled plastome using Circlator v1.5.5 (Hunt et al. 2015) and SAMtools 1.9 (Li et al. 2009). Genome annotation was initially performed using CPGAVAS2 (Shi et al. 2019), with *Petunia exserta* (MT644125) as a reference. Gene annotations were subsequently manually corrected in Apollo (Misra and Harris 2005) and further validated using CPGView (Liu et al. 2023). All gene annotations were compared with reference plastomes and manually inspected to ensure accurate gene boundaries. The circular map of the plastome was generated with OGDRAW (Greiner et al. 2019).

### 2.3 Comparative Plastome Analyses

Overall GC content was calculated using seqkit (Shen et al. 2016). The plastomes of *R. chilensis* , *S. sinuata*, and three other Solanaceae species ( *P. exserta* , *B. patagonica* , and *Solanum lycopersicum* ) were compared in mVISTA (Frazer et al. 2004). Whole‐genome alignments were performed using the shuffle LAGAN algorithm implemented in mVISTA, with *R. chilensis* as a reference sequence. The connecting regions of the SC and IR regions were visualized and compared using CPJSdraw v0.0.1 (Li et al. 2023).

### 2.4 Codon Usage and Repeat Sequence Analyses

Codon usage patterns for protein‐coding genes in the cp genomes of *R. chilensis* and *S. sinuata* were investigated. The relative synonymous codon usage (RSCU) values were calculated, which measure the observed frequency of a codon relative to the expected frequency under equal synonymous codon usage (Sharp and Li 1986; Raman et al. 2019). An RSCU value > 1.00 indicates preferential codon usage, whereas a value < 1.00 indicates that a codon is used less frequently than expected. Codon usage distributions and amino acid frequencies were visualized in R v4.1.1.

Simple sequence repeats (SSRs) were identified using MISA (Beier et al. 2017). The minimum repeat thresholds were set to 10, 5, 4, and 3 repeat units for mono‐, di‐, tri‐, and tetra−/penta−/hexanucleotide motifs, respectively. Dispersed repeats, including forward and palindromic repeats, were detected using Vmatch (Kurtz et al. 2001), with a minimum repeat size of 30 bp and a Hamming distance of 3.

### 2.5 Phylogenetic Analysis

To infer phylogenetic relationships, the newly sequenced cp genomes of *R. chilensis* and *S. sinuata* were analyzed together with 34 representative plastid genomes from diverse lineages of the Solanaceae. *Ipomoea alba* (ON209203.1) was selected as the outgroup taxon. Complete plastome sequences were aligned by MAFFT v.7 (Katoh and Standley 2013) under default settings. Phylogenetic reconstruction was performed in IQ‐TREE v2.0 (Nguyen et al. 2014), and branch support was evaluated using 1000 ultrafast bootstrap replicates (Hoang et al. 2018).

### 2.6 Nucleotide Diversity Analyses

The 36 Solanaceae cp genomes used in phylogenetic reconstruction were further comparatively analyzed. Shared protein‐coding genes and intergenic spacer regions were extracted using Phylosuite (Zhang et al. 2020). The gene and intergenic region matrices were manually inspected in BioEdit (Hall et al. 2011). For duplicated genes with identical sequences within the same species, only a single copy was retained. For intergenic regions, only those containing sequences from more than 19 species were kept, resulting in a final dataset of 59 intergenic spacers for subsequent analyses. The nucleotide sequences of each gene and intergenic region were aligned by MAFFT (Katoh and Standley 2013). Nucleotide Diversity (Pi) for each DNA region was subsequently calculated using DnaSP (Rozas et al. 2017) to identify highly variable regions.

## 3 Results and Discussion

### 3.1 Sequencing of *R. chilensis* and *S. sinuata*

A total of 34.81 million and 40.10 million clean Illumina sequencing reads were obtained for *R. chilensis* and *S. sinuata* , respectively. Mapping of clean reads back to the assembled plastomes revealed average sequencing depths of 2089× for *R. chilensis* and 432× for *S. sinuata* . The high read coverage of both cp genomes provided strong support for the high reliability and accuracy of the plastome assemblies.

### 3.2 General Features of Chloroplast Genomes and Comparison With Related Species

The assembled circular plastomes of *R. chilensis* (156,969 bp) and *S. sinuata* (156,262 bp) exhibit the characteristic quadripartite pattern commonly observed in angiosperms (Figure 1). It comprises an LSC region of 87,459 bp ( *R. chilensis* ) and 86,669 bp ( *S. sinuata* ), a SSC region of 18,422 bp ( *R. chilensis* ) and 18,601 bp ( *S. sinuata* ), and a pair of IR regions (IRa and IRb), each 25,544 bp ( *R. chilensis* ) and 25,496 bp ( *S. sinuata* ), respectively. The chloroplast genome comprised 112 (111 in *S. sinuata* ) unique genes, including 79 protein‐coding genes, 29 (28 in *S. sinuata* ) tRNA genes, and 4 rRNA genes (Table 1). Twenty‐two genes contained introns, including 14 genes with a single intron and three genes (*rps12*, *ycf3*, and *clpP*) containing two introns (Table 1). Overall, gene content and genome organization were nearly identical between the two species.

**FIGURE 1:**
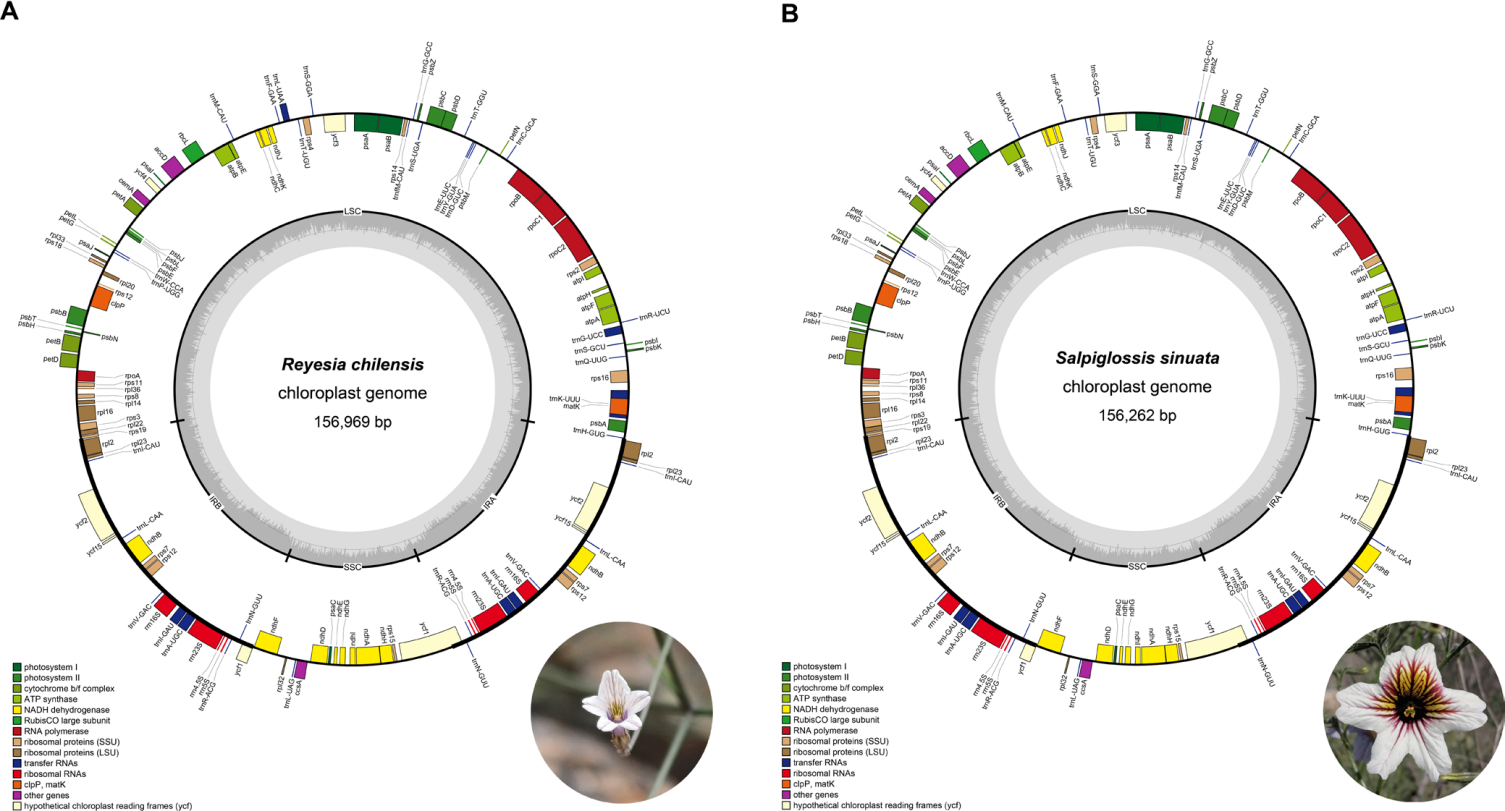
Circular maps of the cp genomes of *R. chilensis* (A) and *S. sinuata* (B). The map illustrates the quadripartite organization comprising an LSC region, an SSC region, and two IR regions (IRa and IRb). Genes positioned on the inner side of the circle are transcribed in a clockwise direction, whereas those on the outer side are transcribed counterclockwise. Different colors denote functional categories of annotated genes, while the inner circle displays the genomic GC content in dark gray and AT content in light gray. Photo credits: *R. chilensis* (photograph by Álvaro Parra Valdivia) and *S. sinuata* (photograph by victorbustos), sourced from iNaturalist. Photos are not to scale.

**TABLE 1:** Plastome gene composition of *R. chilensis* and *S. sinuata* .

Category of genes	Group of genes	Name of genes	Amount
Self‐replication	Ribosomal RNA	*rrn16S* (x2), *rrn23S* (x2), *rrn5S* (x2), *rrn4.5S* (x2)	8
	Transfer RNA	*trnA*‐UGC*(x2), *trnC*‐GCA, *trnD‐*GUC, *trnE*‐UUC, *trnF*‐GAA, *trnG*‐GCC, *trnG*‐UCC*, *trnH*‐GUG, *trnI*‐CAU (x2), *trnI*‐GAU*(x2), *trnK*‐UUU*, *trnL*‐CAA (x2), *trnL*‐UAA*, ^a^, *trnL*‐UAG, *trnM*‐CAU, *trnN*‐GUU (x2), *trnP*‐UGG, *trnQ*‐UUG, *trnR*‐ACG (x2), *trnR*‐UCU, *trnS*‐GCU, *trnS*‐GGA, *trnS*‐UGA, *trnT*‐GGU, *trnT*‐UGU, *trnV‐*GAC (x2), *trnW*‐CCA, *trnY*‐GUA, *trnfM*‐CAU	36 (35)
	Large subunit of ribosome	*rpl14*, *rpl16* *, *rpl2* * (x2), *rpl20*, *rpl22*, *rpl23*(x2), *rpl32*, *rpl33*, *rpl36*	11
	Small subunit of ribosome	*rps11*, *rps12* * (x2), *rps14*, *rps15*, *rps16* *, *rps18*, *rps19*, *rps2*, *rps3*, *rps4*, *rps7*(x2), *rps8*	14
	DNA dependent RNA polymerase	*rpoA*, *rpoB*, *rpoC1* *, *rpoC2*	4
Photosynthesis	Subunits of ATP synthase	*atpA*, *atpB*, *atpE*, *atpF* *, *atpH*, *atpI*	6
	Subunits of photosystem I	*psaA*, *psaB*, *psaC*, *psaI*, *psaJ*	5
	Subunits of photosystem II	*psbA*, *psbB*, *psbC*, *psbD*, *psbE*, *psbF*, *psbH*, *psbI*, *psbJ*, *psbK*, *psbL*, *psbM*, *psbN*, *psbT*, *psbZ*, *ycf3* *	16
	Subunits of NADH‐dehydrogenase	*ndhA* *, *ndhB* * (x2), *ndhC*, *ndhD*, *ndhE*, *ndhF*, *ndhG*, *ndhH*, *ndhI*, *ndhJ*, *ndhK*	12
	Subunits of cytochrome b/f complex	*petA*, *petB* *, *petD* *, *petG*, *petL*, *petN*	6
	Subunit of Rubisco	*rbcL*	1
Other genes	Subunit of Acetyl‐CoA‐carboxylase	*accD*	1
	c‐type cytochrome synthesis gene	*ccsA*	1
	Envelop membrane protein	*cemA*	1
	Protease	*clpP* *	1
	Maturase	*matK*	1
	Conserves open reading frames	*ycf1*(x2), *ycf15* (x2), *ycf2*(x2), *ycf4*	7
Total number of genes			131 (130)

*Note:* If the number of genes of two species in a certain category is different, the number of genes in *S. sinuata* is indicated in parentheses. (x2) indicates that the gene has two copies.

^a^ Indicates that the gene exists only in *R. chilensis* .

* Represents genes containing introns.

The cp genome sizes of these two species were comparable to those of the closely related species *P. exserta* and *B. patagonica* , whose plastome lengths are 156,597 bp and 155,570 bp, respectively. Similarly, only minor variations were observed in the sizes of the four chloroplast genome regions among these species (Table 2), with the latter two species possessing LSC regions of 87,095 bp and 86,085 bp, SSC regions of 18,642 bp and 18,445 bp, and IR regions of 25,430 bp and 25,520 bp, respectively.

**TABLE 2:** Genome comparisons of *R. chilensis* , *S. sinuata* , and other related species.

Species		*R. chilensis*	*S. sinuata*	*P. exerta*	*B. patagonica*
Genome length (bp)		156,969	156,262	156,597	155,570
LSC length (bp)		87,459	86,669	87,095	86,085
SSC length (bp)		18,422	18,601	18,642	18,445
IR length (bp)		25,544	25,496	25,430	25,520
GC content (%)	Total	37.88	37.93	37.81	37.79
	Large‐single copy	35.96	36.04	35.93	35.86
	Small‐single copy	32.24	32.19	32.07	31.78
	Inverted repeats	43.19	43.25	43.14	43.21
Accession Number		PZ281873	PZ435423	MT644125	PV928260

Comparative analyses involving *R. chilensis* , *S. sinuata* , *P. exserta* , *B. patagonica* , and *S. lycopersicum* revealed extensive conservation of plastome structure and gene composition across the examined Solanaceae species (Figure 2). Most protein‐coding genes (e.g., *rpo* genes, *atp* genes, and *matK*, etc.) as well as several tRNA genes were distributed in the LSC region, whereas the SSC region mainly contained *ndh* genes. Whole‐plastome alignments indicated that different levels of sequence divergence were distributed throughout the genomes. Most variations were located in LSC and SSC regions (Figure 2), while the IR regions remained relatively conserved. Among the five species examined, *R. chilensis* and *S. sinuata* exhibited the highest level of sequence similarity (Figure 2), reflecting their close evolutionary relationship. Most variable sites were distributed in conserved non‐coding sequences (CNS) regions. Nevertheless, several genes, including *clpP*, *ycf1*, and *cemA*, also showed elevated levels of sequence divergence (Figure 2). Detailed quantitative assessments of nucleotide diversity are presented in Section 3.6.

**FIGURE 2:**
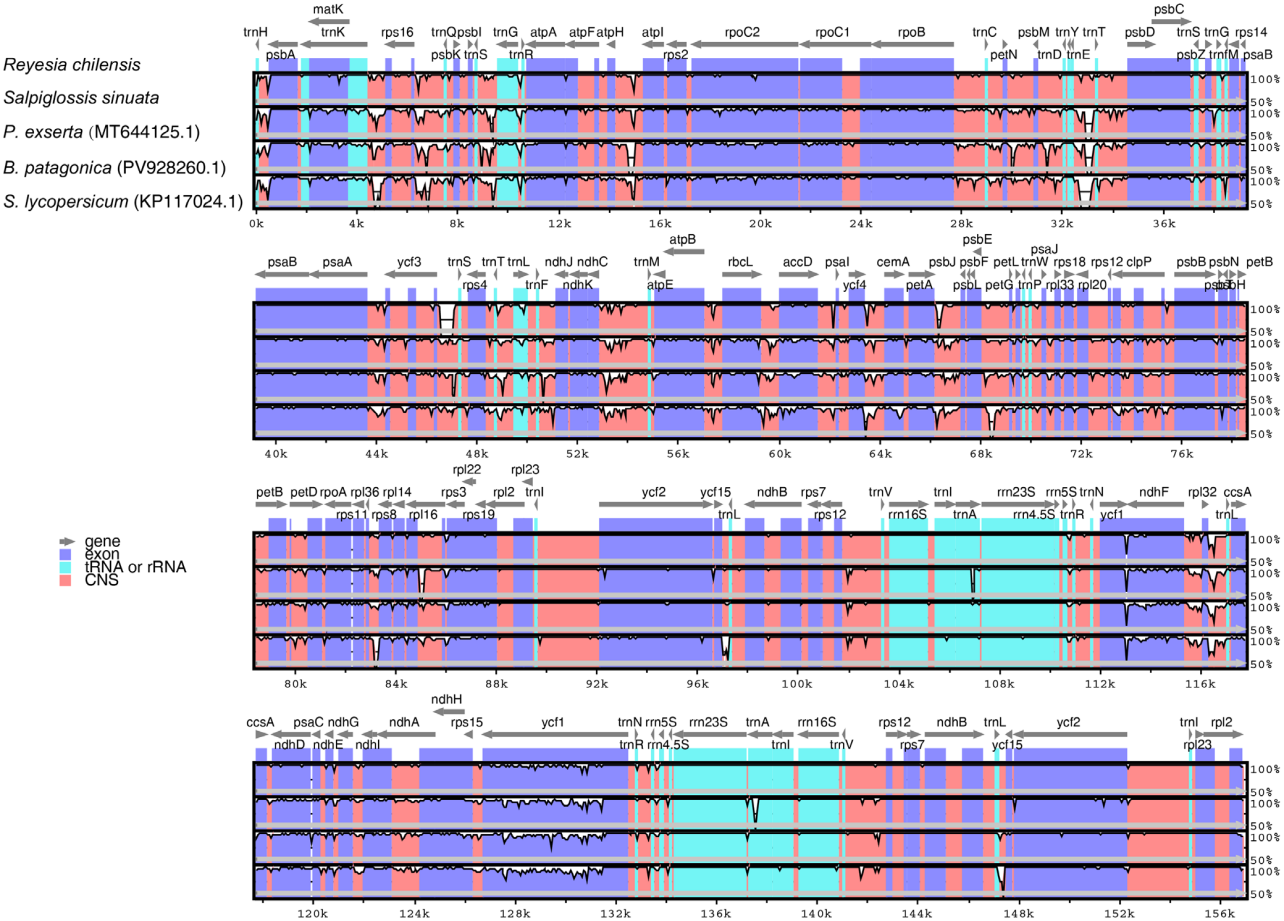
Comparison of the plastomes of *R. chilensis* , *S. sinuata* , and three other Solanaceae species using mVISTA. Genes and their directions are marked by gray arrows above the aligned sequences. Different genomic regions are represented by distinct colors. The vertical scale of each sequence represents the percentage of sequence similarity among species.

Comparisons of the four junctions between the single‐copy and inverted‐repeat regions (LSC, SSC, and IR) revealed only limited structural variation among representative Solanaceae plastomes (Figure 3). At the LSC/IRb boundary, the *rps19* gene was partially extended into (61–92 bp) the IRb region. At the IRb/SSC boundary, *ycf1* spanned the junction with a small portion (1–21 bp) extending into the IRb, and the *ndhF* gene was entirely located within the SSC region and adjacent to the boundary with a 2–69 bp region. At the SSC/IRa boundary, another *ycf1* crossed the boundary with a fraction of 987–1119 bp, resulting in the truncated pseudogene junction region. At the IRa/LSC boundary, the *trnH* gene was situated within the LSC region, although the distance between the gene and the junction differed slightly among species (1–25 bp). These observations indicate that plastome structure was highly stable across Solanaceae, with only minor expansions and contractions contributing to genome‐length variation.

**FIGURE 3:**
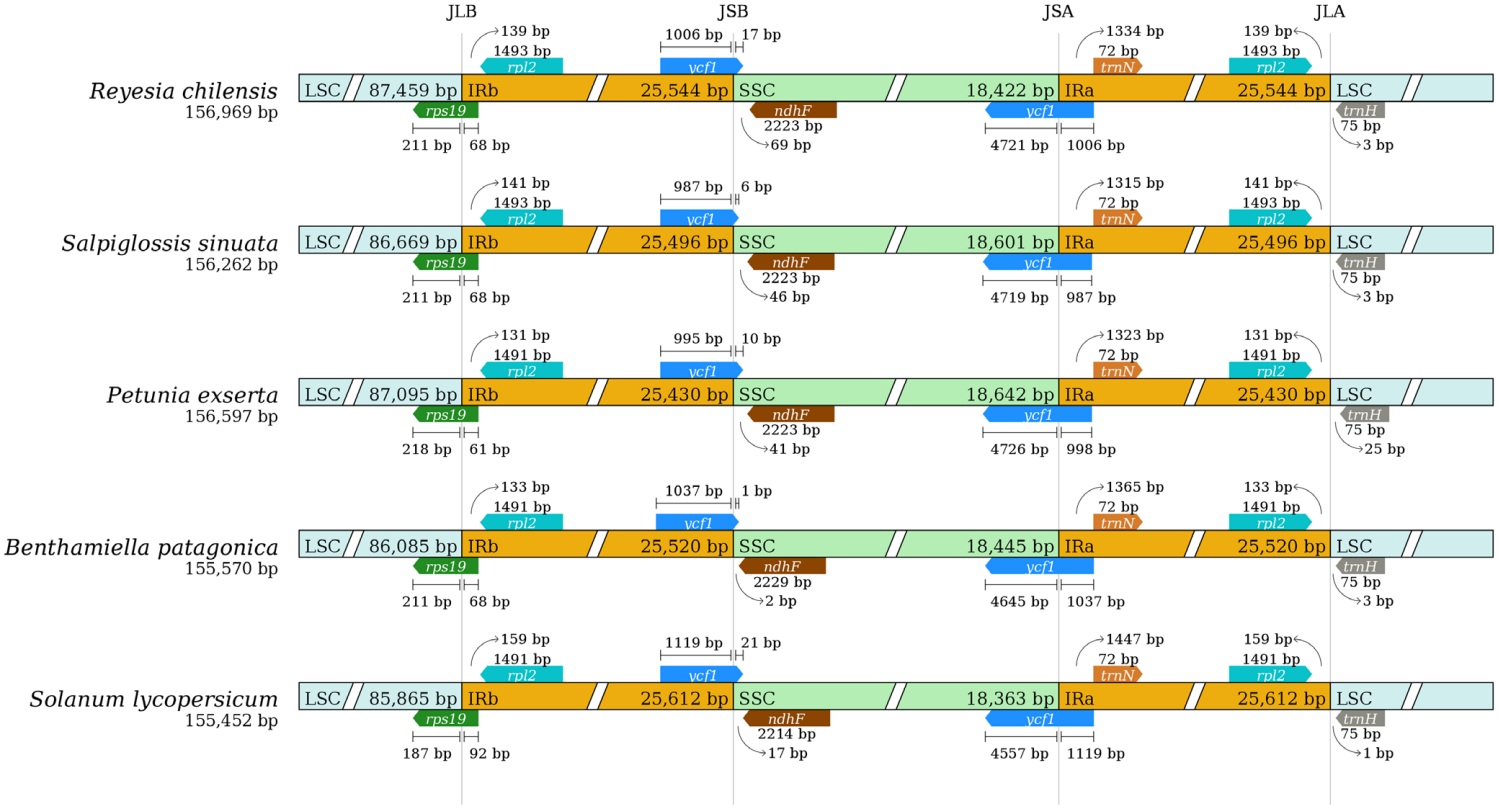
Comparative analysis of gene organization among representative Solanaceae plastomes.

The plastomes of *R. chilensis* and *S. sinuata* conform to the highly conserved structural pattern widely reported across Solanaceae and other angiosperm species (Nguyen et al. 2020; Lei et al. 2021; Wang et al. 2022; Huang, Lu, et al. 2023; Yisilam et al. 2025). Their genome sizes, gene content, and gene organization closely resemble those of numerous Solanaceae taxa, such as *Capsicum* and Physalideae (He et al. 2024; Sandoval‐Padilla et al. 2025). The presence of about 79 protein‐coding genes, approximately 30 tRNA genes, and four rRNA genes represents the most common gene composition pattern reported in most published plastomes (Sandoval‐Padilla et al. 2025; Xu et al. 2025). Although plastome evolution within Solanaceae is generally conservative, lineage‐specific gene losses have occasionally been reported. For example, complete loss of both copies of *ycf15* has been documented in *Archiphysalis* and several species of *Nicotiana* (Wang et al. 2022; Xu et al. 2025). Nevertheless, no substantial gene loss or structural rearrangement was detected in either *R. chilensis* or *S. sinuata* , further supporting the remarkable conservation of plastome organization within these two species.

### 3.3 Simple Sequence Repeats and Dispersed Repeats

A total of 53 SSRs were identified in each of the two plastomes (Figure 4A). Mononucleotide repeats constituted the largest proportion of SSRs (50.94%, *n* = 27 in both *R. chilensis* and *S. sinuata* ), followed by dinucleotide (18.86%, *n* = 10 in both species), trinucleotide (3.77%–5.66%, *n* = 2/3 in *S. sinuata* / *R. chilensis* , respectively), and tetranucleotide (24.5%, *n* = 13 in both species). Only a single pentanucleotide repeat was identified, occurring exclusively in *S. sinuata* . The overall SSR composition was highly similar between the two species, reflecting the conserved pattern of their plastid genomes. Most mononucleotide SSRs were composed of poly A or T motifs, indicating a pronounced enrichment of A/T repeats. In addition to SSRs, 25 and 28 dispersed repetitive sequences were identified in *R. chilensis* and *S. sinuata* , respectively (Figure 4B). *R. chilensis* contained 13 forward and 12 palindromic repeats, whereas *S. sinuata* possessed 14 repeats of each category. Most dispersed repeats had lengths ranging from 30 to 50 bp, with only a few exceeding this size range.

**FIGURE 4:**
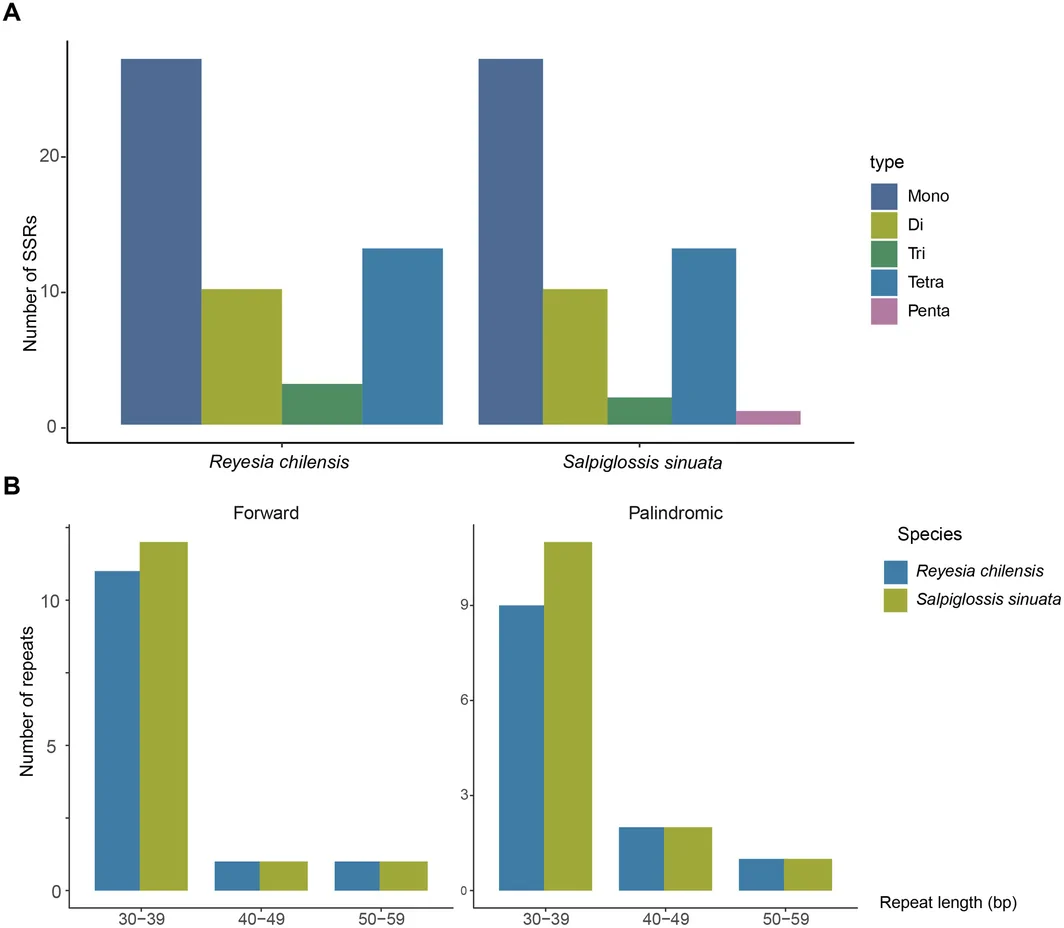
Comparison of SSRs and dispersed repeats in the plastomes of *R. chilensis* and *S. sinuata* . (A) Types and numbers of SSRs. The *x*‐axis represents the detected SSR categories, and the *y*‐axis indicates the number of each SSR type. (B) Comparison of dispersed repeats. Forward and palindromic repeats are presented separately, with repeat abundance summarized according to repeat size classes.

A/T repeats were the most abundant mononucleotide SSRs, reflecting the strong A/T bias of the chloroplast genome. This bias of A/T‐rich SSR motifs is consistent with patterns reported in other angiosperm cp genomes and is considered to contribute substantially to the overall GC content of plastomes (Bessega et al. 2013; Gichira et al. 2019; Li et al. 2021). Length polymorphisms of SSRs have proven valuable for species delimitation and genetic diversity assessments in many angiosperm groups (Singh et al. 2018; Zhang et al. 2023). Although no major substantial changes were detected in the plastomes of *R. chilensis* and *S. sinuata* , the abundance of SSRs and dispersed repeats provided useful resources for future population genetics, phylogeographic investigations, and the development of molecular markers in Solanaceae.

### 3.4 Codon Usage Patterns and Amino Acid Composition

Analysis of the RSCU of protein‐coding genes in these three species revealed a strong preference for codons terminating in A or T in both plastomes (Figure 5A). Among codons encoding leucine (Leu), TTA (RSCU = 1.92/1.90 in *R. chilensis* / *S. sinuata* , respectively) has a higher preference compared with CTG (RSCU = 0.39 in both species), reflecting the overall AT‐rich pattern of the plastid coding sequences. RSCU values greater than 1 indicate a preferential codon for an amino acid, whereas a value less than 1 indicates less frequent usage. Across the two species, 30 codons have RSCU values exceeding 1.00 (Figure 5A), indicating a consistent pattern of codon preference.

**FIGURE 5:**
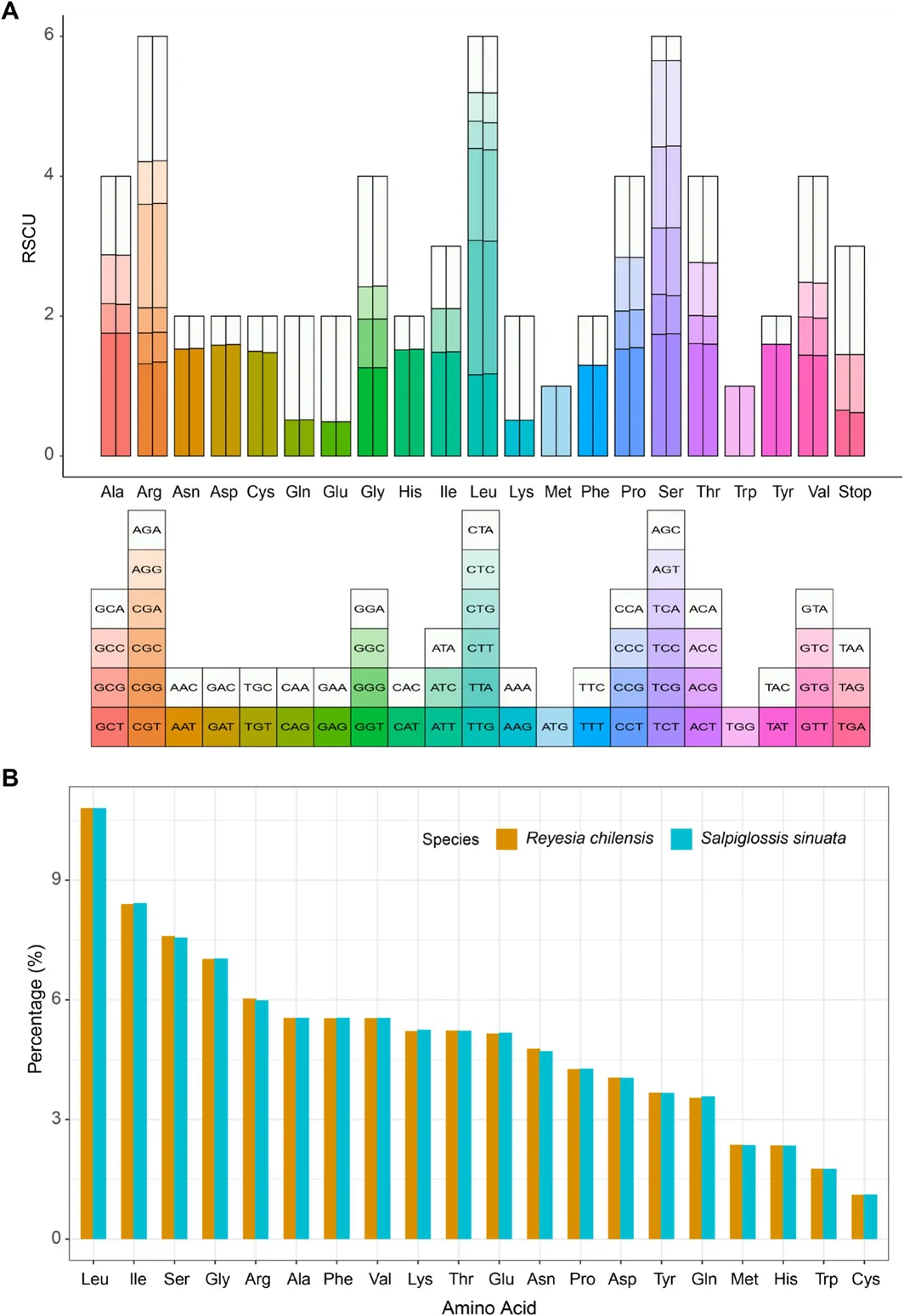
Codon usage patterns and amino acid composition of protein‐coding genes. (A) RSCU for all synonymous codons. Amino acids are arranged along the x‐axis, and each stacked bar represents the codons encoding a given amino acid. (B) Amino acid composition. The *Y*‐axis indicates the proportion of each amino acid relative to the total amino acid content of the plastome.

Substantial variation in amino acid frequencies was observed across the chloroplast genomes (Figure 5B), yet consistently followed a pattern in which leucine was the predominant amino acid (Leu, 10.81% in both species), followed by isoleucine (Ile, 8.40% and 8.43% in *R. chilensis* and *S. sinuata* , respectively), serine (Ser, 7.60% and 7.56%), and glycine (Gly, 7.02% and 7.04%). In contrast, tryptophan (Trp, 1.76% in both species) and cysteine (Cys, 1.10% and 1.11% in *R. chilensis* and *S. sinuata* , respectively) are the least frequently occurring amino acids.

Codon usage bias has been widely employed to investigate gene evolution, phylogenetic relationships, and environmental adaptation among plant lineages (Athey et al. 2017). The predominance of Leu, Ile, and Ser observed here is consistent with patterns reported in numerous Solanaceae plastomes as well as across diverse angiosperm groups (He et al. 2024; Abdullah et al. 2025; Xu et al. 2025). Likewise, the strong preference for A/T‐ending codons reflects the AT‐rich composition characteristic of plastid genomes (Gichira et al. 2019; Raman et al. 2019; Wang et al. 2021; Liu et al. 2022). This highly conserved codon usage and amino acid profiles observed in these taxa suggest that similar evolutionary pressures have shaped chloroplast genome evolution throughout Solanaceae and perhaps across land plants more broadly (Abdullah et al. 2025; Wang et al. 2026).

### 3.5 Phylogenetic Placement of *R. chilensis* and *S. sinuata*

Our plastome‐based phylogenetic analysis resolved the relationships among several subfamilies within Solanaceae with strong bootstrap support (BS) across major nodes (Figure 6). The earliest diverging clade comprised Cestroideae ( *R. chilensis* and *S. sinuata* ) and Benthamielleae (*Benthamiella patagonica*). The remaining major lineages included Petunioideae, Nicotianoideae, and Solanoideae. Among these, Solanoideae represented the most species‐rich lineage, comprising major groups such as Hyoscyameae (*Hyoscyamus* Tourn. ex L., *Atropa* L., etc.), Physalideae (*Physalis* L., *Withania* Pauquy, etc.), Capsiceae (*Capsicum*), and Solaneae (*Solanum* and *Jaltomata* Schltdl.). These results are largely consistent with previous nuclear phylogenetic analyses (Huang, Xu, et al. 2023), indicating that complete plastome sequences also contain substantial phylogenetic information to resolve relationships within Solanaceae.

**FIGURE 6:**
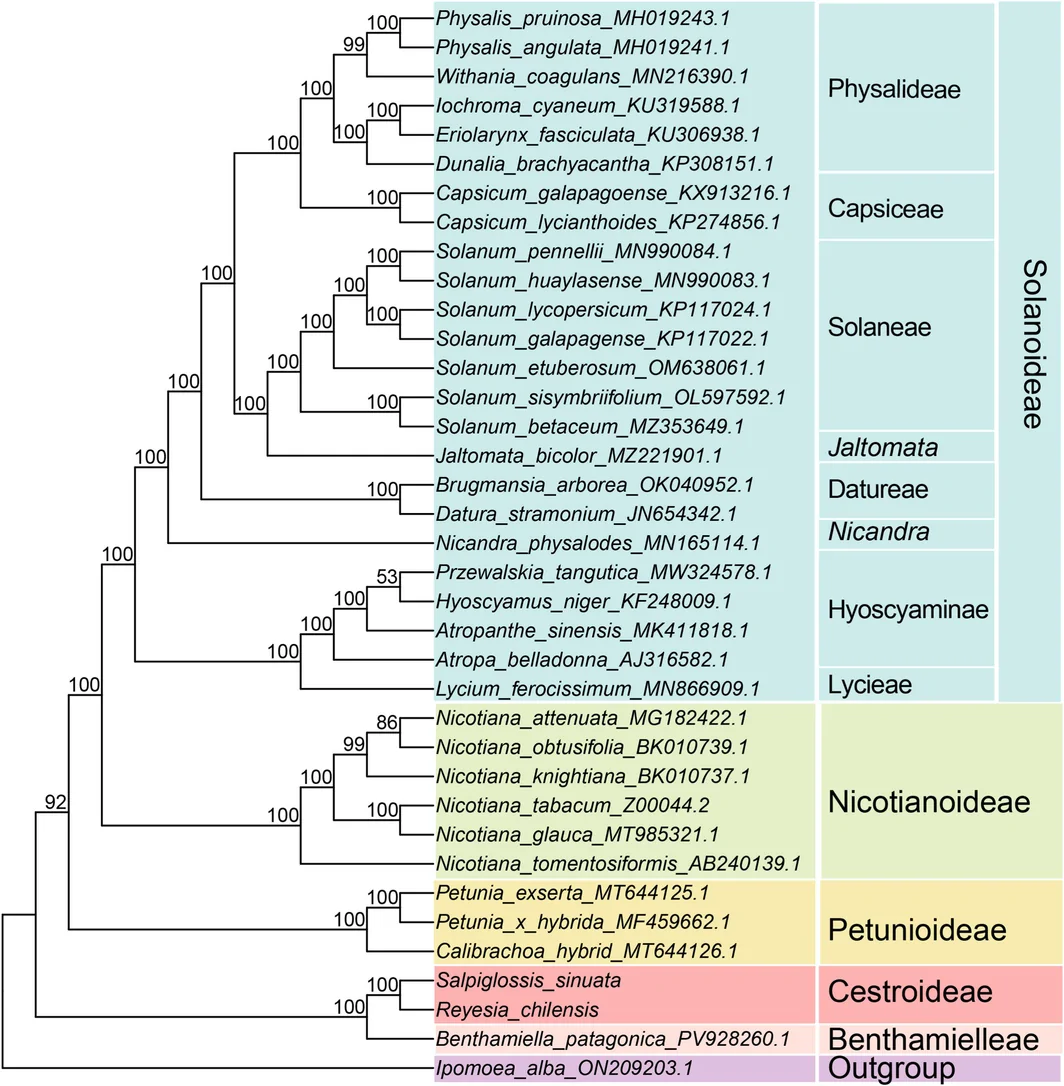
The phylogeny of *R. chilensis* and *S. sinuata* with other Solanaceae species. The tree was reconstructed utilizing complete cp genome data and rooted by *Ipomoea alba* as the outgroup. Species are highlighted with distinct colored backgrounds to indicate their respective subfamilies or clades, with their names shown on the right.

The phylogenetic position of *Benthamiella patagonica* has long remained uncertain, with the genus treated as *incertae sedis* within Solanaceae (Särkinen et al. 2013; Barboza et al. 2016; Deanna et al. 2026). A recent plastome‐based phylogenetic analysis placed it as the deepest‐diverging lineage within the family (Abdullah et al. 2025). However, this study lacked representatives from several early‐diverging Solanaceae lineages, such as Cestroideae (*Reyesia*, *Salpiglossis*, *Cestrum* L., *Browallia*, etc.), Schizanthoideae (*Schizanthus*), and Goetzeoideae (*Goetzea*) (Särkinen et al. 2013; Ng and Smith 2016; Dupin et al. 2017; Huang, Xu, et al. 2023; Deanna et al. 2026). By incorporating newly sequenced plastomes from *R. chilensis* and *S. sinuata* , the present study fills this important sampling gap. Our analyses recovered *R. chilensis* and *S. sinuata* as a strongly supported sister group (BS = 100%), which was subsequently clustered with *B. patagonica* (BS = 100%), together representing the earliest‐diverging lineage within Solanaceae (Figure 6).

The taxonomic placement of *Reyesia* has also varied among previous classifications and phylogenetic studies. Most analyses based on plastid markers have recovered it as closely related to the morphologically similar genus *Salpiglossis* Ruiz & Pav (Olmstead and Bohs 2007; Ng and Smith 2016; Deanna et al. 2026), whereas alternative hypotheses have placed *Reyesia* with Goetzeoideae and *Duckeodendron* Kuhlm. as part of the earliest‐diverging lineage of Solanaceae (Särkinen et al. 2013; Dupin et al. 2017). Our plastome analysis supports the close relationship between *Reyesia* and *Salpiglossis*, consistent with their shared morphological characteristics. Both genera are herbaceous plants characterized by zygomorphic flowers and capsular fruits, whereas members of Goetzeoideae and *Duckeodendron* are woody taxa having actinomorphic flowers and fleshy fruits (Barboza et al. 2016). The concordance between these morphological similarities and the plastome topology, together with the close relationship reported in previous nuclear phylogenomic studies (Huang, Xu, et al. 2023), supports the recognition of *Reyesia* and *Salpiglossis* as closely related members of Salpiglossideae. Although the present study fills an important gap in plastome sampling, several early‐diverging branches of Solanaceae remain insufficiently represented by complete chloroplast genomes. Expanded sampling of these lineages (e.g., Schizanthoideae, Goetzeoideae, etc.) will be essential for refining deep phylogenetic relationships and improving comparative evolutionary analyses across the family.

Despite general agreement, plastome and nuclear phylogenies may reflect different aspects of evolutionary history and should not be assumed to be fully congruent. For example, plastid‐based analyses placed *Jaltomata* as the sister group of *Solanum* in our analysis (Figure 6) and previous studies (Olmstead et al. 2008; Särkinen et al. 2013; Ng and Smith 2016), whereas nuclear phylogenomic analyses supported an alternative placement of *Jaltomata* as sister to Physalideae or Capsiceae (Wu et al. 2019; Huang, Xu, et al. 2023). This contrast illustrates that plastid and nuclear datasets can yield different topological hypotheses for the same lineage. Because the plastome constitutes a single, typically uniparentally inherited genomic compartment, plastome‐based phylogenies primarily reflect the evolutionary history of an organellar lineage and may therefore differ from relationships inferred from nuclear genomes, which incorporate genetic contributions from both parents. Potential explanations include incomplete lineage sorting, introgression or hybridization, and chloroplast capture, which are not mutually exclusive (Tomlin et al. 2024; Peng et al. 2025). Similar cytonuclear conflicts have been documented in other rapidly diversifying Solanaceae lineages, particularly *Solanum*, where incomplete lineage sorting, polyploidization, and reticulate evolution might have contributed to complex phylogenetic patterns (Gagnon et al. 2022; Winslow et al. 2026).

### 3.6 Nucleotide Diversity and Candidate Molecular Markers

To evaluate sequence divergence across Solanaceae plastomes, nucleotide diversity was estimated for 79 protein‐coding genes and 59 intergenic regions shared among the 36 sampled species. Coding regions exhibited relatively low levels of variation, with an average Pi of 0.013 (Figure 7A). The ten most divergent sequences, ranked by Pi value, were *clpP* (Pi = 0.064), *ycf1* (0.044), *cemA* (0.039), *rpl32* (0.030), *rpl22* (0.028), *matK* (0.025), *ndhF* (0.023), *psbT* (0.023), *ccsA* (0.023), and *rpl20* (0.022) (Figure 7A). Nine genes were found to be highly conserved, with Pi values lower than 0.005, including *psaJ*, *rpl2*, *ycf15*, *psbF, rpl23*, *psbL*, *psbN*, *ndhB*, and *rps7* (Figure 7A). Intergenic regions showed greater variability than coding sequences, with an average Pi value of 0.028 (Figure 7B). The most variable spacers included *trnH‐psbA* (0.077), *trnL‐trnF* (0.075), *rpl32‐trnL* (0.057), *rpl36‐rps8* (0.052), *rps15‐ycf1* (0.050), *ccsA‐ndhD* (0.048), *petA‐psbJ* (0.044), *rps16‐trnQ* (0.043), *trnM‐atpE* (0.042), and *trnE‐trnT* (0.041). Several intergenic regions exhibited extremely limited sequence variation with Pi values below 0.005 (*rrn4.5S*‐*rrn5S*, *ndhB‐rps7*, *rps7‐trnV*, *rnV‐rrn16*, *rrn16‐trnI*).

**FIGURE 7:**
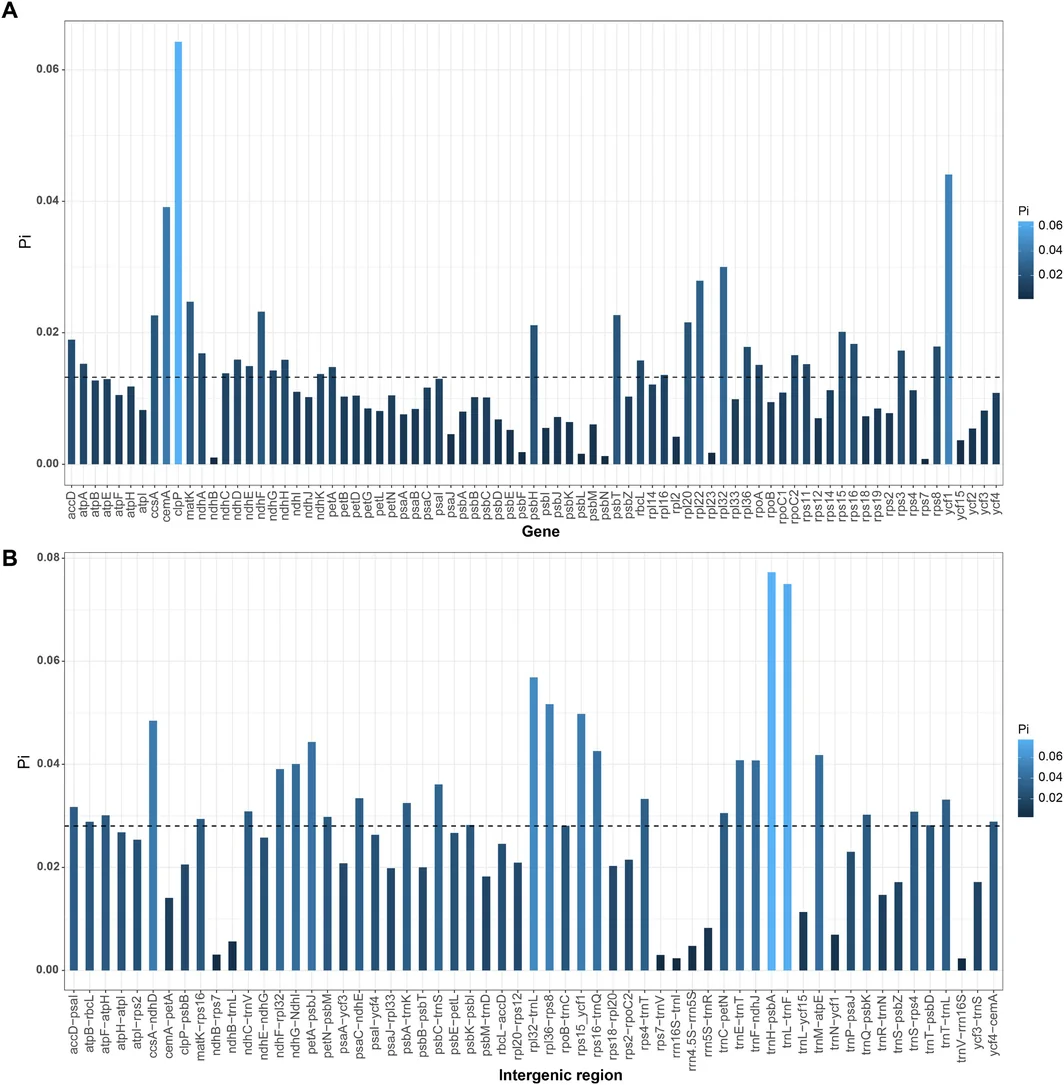
Sequence polymorphism of shared plastid genes of Solanaceae species. Bars represent Pi values for individual loci, with bar colors ranging from dark blue to light blue, corresponding to low to high Pi values, respectively. (A) Pi of 79 shared genes. The dashed horizontal line indicates the average Pi value (0.013). (B) Pi of intergenic regions. The average Pi value (0.028) is indicated by a dashed line.

Protein‐coding regions of plastomes are generally highly conserved in angiosperm plants (Daniell et al. 2016; Dobrogojski et al. 2020). Many chloroplast loci identified as highly variable in the present study correspond to markers that have already proven useful in Solanaceae phylogenetics. For example, *ndhF* and *trnT–trnF* were used to reconstruct the phylogeny of *Solanum* (Weese and Bohs 2007) and Solanaceae (Olmstead et al. 2008). Subsequently, additional chloroplast loci have been incorporated into phylogenetic analyses of the family or specific lineages, such as *matK*, *psbA–trnH*, and *trnS–trnG* (Särkinen et al. 2013; Carrizo Garcia et al. 2016; Ng and Smith 2016). Other plastid markers not commonly used in Solanaceae research practice included sequences from *atpB*, *rps16–trnK*, *rbcL*, *trnC*–*psbM*, *trnD*–*trnT*, *rpl32*–*trnL*, and *ndhF*–*rpl32* (Tu et al. 2010; Moré et al. 2015). The elevated nucleotide diversity observed in loci such as *clpP*, *cemA* (Figure 7) suggests that these regions may provide additional phylogenetic information for resolving relationships among closely related taxa. These plastid regions with significant variation have also been identified in other angiosperms (Liu et al. 2022). Several genes (such as *ycf1*, *matK*, and *ndhF*) or intergenic regions (*trnH‐psbA* and *trnL‐trnF*) have been widely utilized as common markers for constructing phylogenetic relationships among angiosperms (Hollingsworth et al. 2009; China Plant et al. 2011; Li, Liu, et al. 2011; Li, Gao, et al. 2011; Dong et al. 2012; Xu et al. 2015). Some DNA markers (such as *matK* and *trnH‐psbA*) have been regarded as effective DNA barcodes for plant identification and classification, allowing for rapid identification of plants (Hollingsworth et al. 2009; Li, Gao, et al. 2011).

## 4 Conclusion

This study presents the first complete chloroplast genomes of *Reyesia chilensis* and *Salpiglossis sinuata* , representing the tribe Salpiglossideae within the early‐diverging subfamily Cestroideae. Comprehensive analyses across Solanaceae species showed a high degree of conservation in plastome structure, gene content, repeat composition, and codon usage. Incorporation of these newly generated plastomes improves taxonomic representation within the family and provides additional evidence for the placement of Salpiglossideae among the earliest‐diverging Solanaceae lineages. Furthermore, several highly variable coding and non‐coding regions were identified as promising candidate markers for further phylogenetic reconstruction, DNA barcoding, and species identification. Collectively, these findings provide valuable genomic resources and contribute to a comprehensive understanding of plastome evolution and deep phylogenetic relationships within Solanaceae.

## Author Contributions

**Jie Huang:** resources, data curation, formal analysis, validation, writing – original draft, writing – review and editing, funding acquisition. **Suyun Nong:** resources, formal analysis, writing – original draft, writing – review and editing. **Yancheng Zhang:** resources, formal analysis, writing – original draft, writing – review and editing. **Weibin Xu:** funding acquisition, supervision.

## Conflicts of Interest

The authors declare no conflicts of interest.

## Data Availability

The data generated in this study have been deposited in the National Center for Biotechnology Information (NCBI; https://www.ncbi.nlm.nih.gov/), and the corresponding GenBank accession numbers for the chloroplast genomes are PZ281873 ( *R. chilensis* ) and PZ435423 ( *S. sinuata* ).
